# Muscle, Health and Costs: A Glance at their Relationship

**DOI:** 10.1007/s12603-018-1058-9

**Published:** 2018-06-13

**Authors:** D. M. Mijnarends, Y. C. Luiking, R. J. G. Halfens, S. M. A. A. Evers, E. L. A. Lenaerts, S. Verlaan, M. Wallace, Jos M. G. A. Schols, J. M. M. Meijers

**Affiliations:** 1 School CAPHRI, Department of Health Services Research, Maastricht University, P.O Box 616, 6200 MD Maastricht, the Netherlands; 2 Nutricia Research, Nutricia Advanced Medical Nutrition, P.O. Box 80141, 3508 TC Utrecht, the Netherlands; 3 Trimbos Institute (Netherlands Institute of Mental Health and Addiction), Department of Public Mental Health, Utrecht, the Netherlands; 4 Department of Internal Medicine, Section of Gerontology and Geriatrics, VU University Medical Center, Amsterdam, the Netherlands; 5 School CAPHRI, Department of Family Medicine, Maastricht University, P.O Box 616, 6200 MD Maastricht, the Netherlands

**Keywords:** Muscle, activities of daily living, quality of life, health care costs

## Abstract

**Objective:**

To assess the association between muscle parameters (mass, strength, physical performance) and activities of daily living (ADL), quality of life (QoL), and health care costs.

**Design:**

Cross-sectional Maastricht Sarcopenia Study (MaSS).

**Setting:**

Community-dwelling, assisted-living, residential living facility.

**Participants:**

227 adults aged 65 and older.

**Measurements:**

Muscle mass, hand grip strength and physical performance were assessed by bio-electrical impedance, JAMAR dynamometer and the Short Physical Performance Battery, respectively. Health outcomes were measured by the Groningen Activity Restriction Scale (disability in ADL) and the EQ-5D-5L (QoL). Health care costs were calculated based on health care use in the past three months.

**Results:**

Muscle strength and physical performance showed a strong correlation with ADL, QoL, and health care costs (P<.01); for muscle mass no significant correlations were observed. Regression analyses showed that higher gait speed (OR 0.06, 95%CI 0.01-0.55) was associated with a lower probability of ADL disability. Furthermore, slower chair stand (OR 1.23, 95%CI 1.08-1.42), and more comorbidities (OR 1.58, 95%CI 1.23-2.02) were explanatory factors for higher ADL disability. Explanatory factors for QoL and costs were: more disability in ADL (OR 1.26, 95%CI 1.12-1.41 for QoL; B = 0.09, P<.01 for costs) and more comorbidities (OR 1.44, 95%CI 1.14-1.82 for QoL; B = 0.35, P<.01 for costs).

**Conclusion:**

Lower gait speed and chair stand were potential drivers of disability in ADL. Disability in ADL and comorbidities were associated with QoL and health care costs in community-dwelling older adults. Improving physical performance may be a valuable target for future intervention and research to impact health burden and costs.

**Electronic Supplementary Material:**

Supplementary material is available for this article at 10.1007/s12603-018-1058-9 and is accessible for authorized users.

## Introduction

After 30 years of age, the balance between muscle growth and break down shifts more to degeneration, leading to a net loss of muscle mass and concurrent loss of muscle strength. The age-related loss of muscle mass and function has been defined as sarcopenia, which is now recognized as a geriatric syndrome.

During the past decades, the effects of low muscle mass and poor physical performance on health outcomes, and more recently also on health care costs, have been studied. Previous studies on the association between muscle parameters (1-4) or sarcopenia (5-8) and QoL, disability in activities of daily living (ADL) and health care utilization show inconsistent results. The inconsistent results may partly be explained by the use of different assessment tools (e.g. questionnaires, performancebased instruments), and the populations (level of care dependency, ethnicity) or age groups under study. Furthermore, muscle mass, muscle strength, and physical performance have their own distinct effect on health and economic outcomes (2-5, 9). For example, where Cawthon et al. (5) found that none of the consensus definitions of sarcopenia was associated with an increased risk of hospitalization, gait speed alone was associated with greater health care utilization. In contrast to the study of Cawthon et al. (5), Bianchi et al. (10) did find an association between sarcopenia defined by the EWGSOP and disability, hospitalization and mortality.

Insight in muscle parameters that contribute most to negative health and economic outcomes could help clinicians and policy makers to focus on improving relevant muscle parameters.

The objective of this study was to look at the association between muscle parameters (muscle mass, muscle strength, physical performance) and ADL, QoL, and health care costs.

## Methods

A detailed description of the methodology and in- and exclusion criteria of the Maastricht Sarcopenia Study (MaSS) has been previously published (11, 12). This section highlights the relevant aspects for this article.

### Setting and sample

The cross sectional MaSS study was undertaken in adults ≥ 65 years living: (i) independently at home without additional care, and (ii) at home or in an assisted living facility with professional home care, or (iii) in a residential living facility with additional professional nursing care and/or meal service, in Maastricht area, in the southern part of the Netherlands. Eligibility criteria encompassed: adults ≥65 years with an understanding of the Dutch language, who gave written informed consent. Persons with an implantable cardiac defibrillator/pacemaker, persons in a wheelchair or bedridden, and those suffering from severe active rheumatoid arthritis, post stroke status with evident lingering symptoms, diseases of the nervous system, acute angina pectoris or dementia were excluded, because they would not have been able to perform the physical tests safely.

### Data collection

Participants were recruited between May 2013 and March 2014. The municipality of Maastricht randomly extracted 2448 addresses of adults ≥65 years. An information letter, informed consent form and stamped response envelope were sent to the selected addresses. After receiving the signed consent form, one of the researchers (D.M./E.L.) made a phone call to check for eligibility and a home visit was planned. Data were collected during a single 1–2 hour home visit. A pilot study was performed to test the feasibility of this method of data collection (13). Standardized protocols were used to ensure conformity of data collection. Home visits were performed in the morning because participants had to be in fasting state for the muscle mass measurement. Characteristics of participants were collected through a questionnaire that included among others, age, sex, comorbidities, and living situation i.e. type of care. Height (stadiometer type SECA 213) and weight (scale type SECA 877) were measured with clothes, but without shoes, and BMI calculated as weight/height^2^.

### Muscle measures

The measures used for muscle mass, strength and performance were evaluated as valid and feasible for the measurement of sarcopenia in a community setting (14). Muscle mass was assessed by bio-electrical impedance (BIA Akern Srl, Florence, Italy 101, 50 kHz) according to the ESPEN guidelines (15). Muscle mass was calculated using the Janssen et al.(16) equation: skeletal muscle mass (kg) = [(height2/BIA analysis resistance×0.401) + (gender×3.825) + (age×–0.071)] + 5.102, where height is in centimeters, resistance in ohms, male gender is coded 1, female 0 and age in years. This equation was developed in a population of 18–86 year olds and is applicable in a Caucasian population, like the Dutch population. Cutoff levels for low muscle mass were based on the calculated skeletal muscle index (SMI): ≤10.75 kg/m^2^ (men) and ≤6.75 kg/ m^2^ (women) (17).

Muscle strength was assessed by a JAMAR hand-held dynamometer (Sammons Preston, Inc, Warrenville, IL). Participants performed one try-out attempt with their arm in 90° angle, followed by three attempts with each hand, alternating left and right. Participants were told to take a deep breath and to start squeezing as they exhaled. Researchers encouraged the participants to squeeze as hard as possible. The maximum grip strength was used in the analyses, with cut-off levels for poor muscle strength defined as <20 kg (women) and <30 kg (men) (18). Physical performance was assessed using the short physical performance battery (SPPB) with a total score ranging from 0–12 (19). The SPPB includes normal gait speed over a 4 meter track (in m/s and score 0–4), 5 times chair stand (in seconds, score 0–4 and dichotomized <17> sec (20)) and a balance test (score 0–4); higher scores indicate better performance. For slow gait speed a cut-off level of ≤0.8 m/s was used (18). The cut-off levels for muscle strength and physical performance and the prevalence of sarcopenia were assessed using the algorithm of the European Working Group on Sarcopenia in Older People (EWGSOP) (18).

### Health and economic outcome measures

Disability in ADL was assessed by the validated Groningen Activity Restriction Scale (GARS) (21). This questionnaire consists of 18 questions with each 4 answer categories ranging from “fully independent without any difficulty” (score 1) to “fully dependent” (score 4). This leads to a total score between 18 and 72, with higher scores indicating more disability in ADL.

The validated EQ-5D-5L questionnaire was used to measure QoL (22). This questionnaire includes questions on mobility, self-care, usual activities, pain/discomfort and anxiety. The scores for each question (1 = no problems at all to 5 = extreme problems) are combined into a health state score (e.g. 11111 for a patient that has no problems at all on any of the five questions) and subsequently converted into an index value (utility score) between 0–1 using a country specific value set (23). The EQ-5D-5L also includes a single question about self-rated overall health, with scores ranging from 0 (worst imaginable health state) to 100 (best imaginable health state).

A detailed description of the cost calculation has been previously published (12). In short, health care utilization was assessed by asking participants about their health care use in the past three months. This made it possible to calculate the costs in the health care sector (e.g. visits to a general practitioner, hospital, paramedical staff) and costs for the patient/family (e.g. medication, purchase of medical aids). Cost prices of health care utilization were obtained from the Dutch costing guideline (24).

### Statistical analyses

Data were analyzed using SPSS version 24 (SPSS Inc, Chicago, IL). Data are presented as means±SD or median (Q1-Q3). Health care costs are presented as means and 95% confidence intervals (CI) using non-parametric bootstrapping with 1000 replications. Differences in characteristics (age, sex, etc.) between (i) older adults living independently at home, (ii) older adults living at home or in an assisted living facility with professional home care, and (iii) older adults living in a residential living facility were assessed by One- Way ANOVA (normally distributed continuous variables), non-parametric Kruskal Wallis Test (non-normally distributed continuous variables) or χ2 test (categorical variables). In the further analyses, the three groups were analyzed all together. This strategy was chosen for its ability to better study the associations between the muscle parameters and the outcome variables, as including all three groups led to a wider range in ADL scores, QoL and costs. Bivariate correlations between muscle parameters (mass, strength, physical performance; as continuous variables) and outcomes (disability in ADL, QoL, health care costs) are provided using Spearman’s rankcorrelation. Boxplots were used to visualize differences in outcome measures for dichotomized muscle parameters (tested with Mann-Whitney U Test). Two separate logistic regression analyses were performed, with as dependent variables 1) ADL function (low/high) and 2) QoL (low/high). The cut-off levels for low/high are based on the median. First, simple logistic regression analyses were run, with only one independent variable at the time. Second, multiple logistic regression analyses were run, with Model 1: forced entry of muscle mass, muscle strength and physical performance; Model 2: Stepwise model adjusted for age, sex, BMI. Adjusting for MMSE score did not influence the analyses and was therefore not included in the final models. The SPPB was excluded from Model 2 because it includes gait speed and chair stand, which are already included as independent variables. Furthermore, linear regression was used to assess the relation between health care costs (dependent continuous variable, log transformed) and muscle parameters (independent continuous variables). A log transformation was applied because the distribution of health care costs was skewed. Living situation was not included as an independent variable, because living situation can be seen as a proxy variable of health care costs. This would lead to an overcorrection of the models. Models were tested for interaction; no interaction effects were found. To test the robustness of the results, the largest outliers (Resid > 2) were defined in each regression model. As the results remained stable after removal of the outliers, the final analyses presented included all data. A two-sided P value less than .05 was considered significant.

**Table 1:**
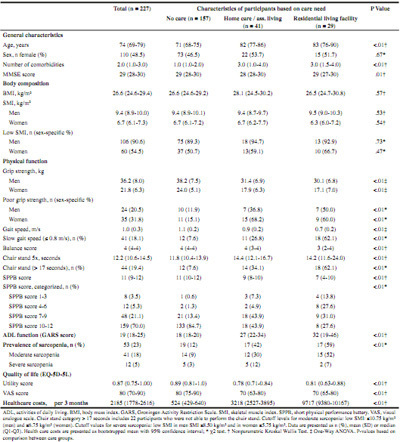
Participant characteristics

**Table 2:**
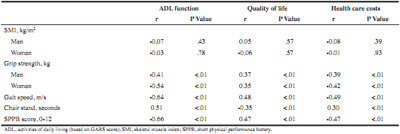
Spearman correlations (r) between muscle, health and economic outcomes (n = 227)

## Results

In total, 227 participants had complete data sets and were included in the analyses.

### Characteristics of the study population

The characteristics of participants are shown in Table 1. Participants in the ‘no care’ group were significantly younger and had fewer comorbidities than participants in the home care and residential living group.

Muscle mass did not differ between care groups, but strength and physical performance were better in the no care group compared with the home care and residential living group. Participants in the home care and residential living group had more disability in ADL (higher GARS scores), a lower QoL and higher health care costs compared with participants in the no care group. Sarcopenia was more prevalent in residential living facilities (59%) compared to those receiving home care (42%) or those living at home without care (12%).

### Muscle versus health outcomes

**Figure 1:**
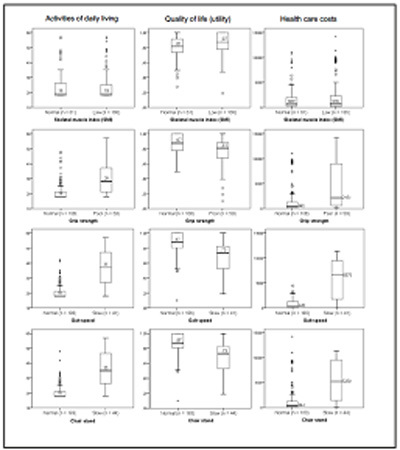
Boxplots representing relation between muscle parameters and ADL, quality of life, and health care costs. Boxplots present the median (middle line) with IQR (boxes), the whiskers represent 1.5 times the IQR, outliers are indicated as circles, extreme outliers with a star

No significant correlations were found between muscle mass with ADL and QoL, but significant correlations with muscle strength and physical performance parameters were found (Table 2). The strongest correlations (r > 0.5) were found between muscle strength and physical performance with ADL. Subscales of ADL, i.e. basic ADL and instrumental ADL, showed the same trend. Most correlations between muscle strength and physical performance and ADL and QoL remained significant when running bivariate correlations in the no care group only (Supplementary Table 1). The ADL and QoL boxplots (Figure 1) show significant differences (P < .01) between groups (normal/low for muscle parameters) for all combinations shown, except for muscle mass versus ADL (P = .23). This indicates that there were no significant differences in ADL function of participants with low SMI versus normal SMI, but participants with poor grip strength, slow gait speed or chair stand had significantly lower ADL function. Participants with low SMI had a significantly higher QoL than participants with normal SMI, but participants with poor grip strength, slow gait speed or chair stand had a significantly lower QoL.

**Table 3:**
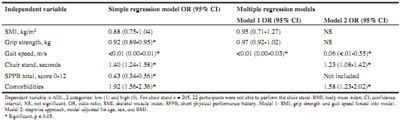
Logistic regression: explanatory factors for activities of daily living (total group, n = 227)

Logistic regression analyses showed that higher gait speed (OR 0.06, 95%CI 0.01-0.55) was associated with a lower probability of physical disability. Furthermore, slow chair stand (OR 1.23, 95%CI 1.08-1.42), and more comorbidities (OR 1.58, 95%CI 1.23-2.02) were significant explanatory factors for higher ADL disability (Table 3). More disability in ADL (OR 1.26, 95%CI 1.12-1.41) and more comorbidities (OR 1.44, 95%CI 1.14-1.82) were significant explanatory factors for QoL (Table 4). For ADL as an example, this means that every extra point on the GARS scale explained 26% higher odds of having a low QoL.

**Figure 2:**
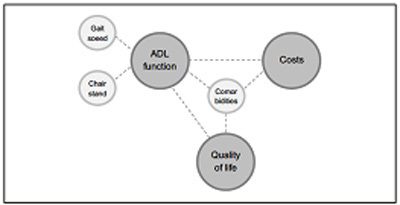
Schematic presentation of associations found between muscle, health and costs

### Muscle versus costs

Muscle strength and physical performance showed significant correlations with health care costs, while no significant correlations were observed for muscle mass (Table 2). When running bivariate correlations in the no care group, none of the muscle parameters was significantly correlated with health care costs (Supplementary Table 1). The boxplots (Figure 1) show significant differences (P < .01) between groups (normal/low) for all combinations of muscle parameters vs. costs, except for muscle mass versus health care costs (P = .90). This indicates that there were no significant differences in health care costs of participants with low SMI versus normal SMI. However, participants with poor grip strength, slow gait speed or chair stand had significantly higher health care costs (P < .01). Linear regression analyses showed that more disability in ADL (B = 0.09, P < .01) and more comorbidities (B = 0.35, P < .01) were explanatory factors for health care costs (Table 5). This means that every 1 point increase in GARS score explained 9% more cost and every (extra) comorbidity explained 35% more cost.

**Table 4:**
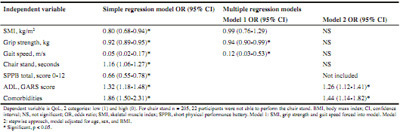
Logistic regression: explanatory factors for quality of life (n = 227)

**Table 5:**
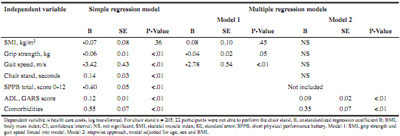
Linear regression: explanatory factors for health care costs (n = 227)

## Discussion

The results of this study show that lower physical performance (gait speed and chair stand), but not muscle mass, was associated with more disability in ADL. This indicates that improving gait speed and chair stand could be a target for research and intervention to achieve an improvement in ADL function. In the final models, none of the muscle parameters were significantly associated with QoL or health care costs. Although no direct association was found between muscle parameters and QoL or health care costs, muscle parameters may indirectly influence QoL and health care costs, via their association with ADL function (Figure 2). Both lower QoL and higher health care costs were associated with poor ADL function and a higher number of comorbidities. Clinicians treating community-dwelling patients with comorbidities should therefore be alert on deteriorations in ADL function. Early identification of older patients with slow gait speed or chair stand, especially those with comorbidities, seems important. Subsequent referral to a physical activity intervention program may prevent deterioration of ADL function. It has been shown that physical activity interventions can improve QoL and are cost-effective (25, 26). In addition, Lo et al. (27) showed that a lower skeletal muscle mass index was associated with higher health care costs over 8 years, but high dietary diversity and more physical activity attenuated these effects. Furthermore, nutritional intervention has been shown to improve muscle mass, physical performance and ADL function (28-29), and may therefore be used in the strategy to combat both malnutrition and sarcopenia related negative health outcomes and costs.

In contrast to our expectations, the boxplots showed that participants with low muscle mass had a higher QoL than participants with normal muscle mass. A potential reason for this could be that many participants with low SMI had normal muscle strength and physical performance. Muscle strength and mainly physical performance seem to be stronger related to QoL and therefore may have blurred the differences in QoL found between low and normal muscle mass. Another explanation could be that the cutoff values for low muscle mass included both moderate and severely low muscle mass. Muscle strength and mainly physical performance seem to be stronger related to QoL and therefore may have blurred the differences in QoL found between low and normal muscle mass. Another reason could be that the muscle mass measurement by bioelectrical impedance, which was selected for feasibility reasons, and the cut-off values used might have overestimated or underestimated muscle mass.

The scores for ADL function and QoL found in the current study lie in between scores found in previous studies using the GARS and the EQ-5D (30-32). Previous studies on health care costs associated with sarcopenia mainly focus on hospitalrelated costs, and are therefore not comparable with the results of the current study (7, 33, 34). In our study, hospital costs accounted for a substantial part of the sarcopenia related health care costs, but the main driver of costs was the living situation (12). This may also explain why the correlation between muscle parameters and health care costs was no longer significant when looking at the no care group only. In addition, in the no care group age and comorbidities (both associated with costs) were lower compared with the residential living group, which may further explain the absence of a significant correlation between muscle parameters and health care costs. In general access to health care services is high in the Netherlands. However, in theory it may be possible that those experiencing ADL disability had worse access to medical care than participants without disability. This may have narrowed the differences in costs between those experiencing ADL disability versus those without disability.

It should be noted that due to the cross-sectional nature of this study no conclusions can be drawn on causal relationships. It can be hypothesized that decreased muscle function leads to more disability in ADL, disability in ADL may in turn lead to decreased muscle function. As participants needed to be able to undergo several physical tests during a two-hour home visit and persons in a wheelchair or bedridden were excluded, our sample might have been healthier compared with the general Dutch population. Furthermore, the majority of the sample was community-dwelling without additional care, while a smaller proportion received care at home or in an institution. Studies in other settings may provide different results. The cost calculation was based on health care utilization in the past three months. Future research prospectively studying costs in (sarcopenic) older adults can add to the evidence base.

## Conclusion

Lower gait speed and chair stand are potential drivers of disability in ADL. Disability in ADL and comorbidities were associated with QoL and health care costs in communitydwelling older adults. Improving physical performance may be a valuable target for future intervention and research to impact health burden and costs.

Acknowledgments: We are thankful to all participants of the MaSS study for their enthusiastic participation. We would like to thank the municipality of Maastricht for their support with the recruitment of participants. Furthermore we would like to acknowledge Egbert Biesheuvel for his valuable support with the statistical analyses.

Conflict of interest statement: This work was supported by Nutricia Research, Utrecht, the Netherlands. Study concept and design: Mijnarends, Halfens, Schols, Meijers, Verlaan, Luiking. Data acquisition: Mijnarends, Lenaerts. Statistical analyses and manuscript preparation: Mijnarends. Interpretation of data and critical revising: all. All authors read and approved the final manuscript.

*Ethical Standards: The Medical Ethics Committee of the Academic Hospital Maastricht and Maastricht University approved the MaSS study. The MaSS study was registered at www.clinicaltrials.gov (NCT01820988)*.

## Electronic supplementary material

**Supplementary Table 1** Spearman correlations (r) between muscle, health and economic outcomes in ‘no care’ group (n = 157)

## References

[1] Al Snih S, Markides KS, Ottenbacher KJ, Raji MA (2004). Hand grip strength and incident ADL disability in elderly Mexican Americans over a seven-year period. Aging Clin Exp Res.

[2] Cesari M, Rolland Y, van Abellan Kan G, Bandinelli S, Vellas B, Ferrucci L (2015). Sarcopenia-related parameters and incident disability in older persons: results from the «Invecchiare in Chianti» study. J Gerontol A Biol Sci Med Sci.

[3] Woo T, Yu S, Visvanathan R (2016). Systematic literature review on the relationship between biomarkers of sarcopenia and quality of life in older people. J Frailty Aging.

[4] Cawthon PM, Fox KM, Gandra SR, Delmonico MJ, Chiou CF, Anthony MS (2009). Do muscle mass, muscle density, strength, and physical function similarly influence risk of hospitalization in older adults. J Am Geriatr Soc.

[5] Cawthon PM, Lui LY, McCulloch CE, Cauley JA, Paudel ML, Taylor B (2017). Sarcopenia and health care utilization in older women. J Gerontol A Biol Sci Med Sci.

[6] Hirani V, Blyth F, Naganathan V L, Couteur DG, Seibel MJ, Waite LM (2015). Sarcopenia is associated with incident disability, institutionalization, and mortality in community-dwelling older men: the Concord Health and Ageing in Men Project. J Am Med Dir Assoc.

[7] Sousa AS, Guerra RS, Fonseca I, Pichel F, Ferreira S, Amaral TF (2016). Financial impact of sarcopenia on hospitalization costs. Eur J Clin Nutr.

[8] Woo J, Leung J, Morley JE (2015). Defining sarcopenia in terms of incident adverse outcomes. J Am Med Dir Assoc.

[9] Visser M, Schaap LA (2011). Consequences of sarcopenia. Clin Geriatr Med.

[10] Bianchi L, Ferrucci L, Cherubini A, Maggio M, Bandinelli S, Savino E (2016). The Predictive Value of the EWGSOP Definition of Sarcopenia: Results From the InCHIANTI Study. J Gerontol A Biol Sci Med Sci.

[11] Mijnarends DM, Schols JM, Meijers JM, Tan FE, Verlaan S, Luiking YC (2015). Instruments to assess sarcopenia and physical frailty in older people living in a community (care) setting: similarities and discrepancies. J Am Med Dir Assoc.

[12] Mijnarends DM, Schols JMGA, Halfens RJG, Meijers JMM, Luiking YC, Verlaan S (2016). Burden-of-illness of Dutch community-dwelling older adults with sarcopenia: health related outcomes and costs. Eur Geriatr Med.

[13] Mijnarends D, Meijers J, Halfens R T, Borg S, Luiking Y, Verlaan S (2013). Rationale and design of a cross-sectional study of the prevalence, characterization and health and economic consequences of sarcopenia in community-dwelling older people in the Netherlands [abstract]. J Nutr Health Aging.

[14] Mijnarends DM, Meijers JMM, Halfens R T, Borg S, Luiking YC, Verlaan S (2013). Validity and reliability of tools to measure muscle mass, strength, and physical performance in community-dwelling older people: a systematic review. J Am Med Dir Assoc.

[15] Kyle UG, Bosaeus I D, Lorenzo AD, Deurenberg P, Elia M, Gomez JM (2004). Bioelectrical impedance analysis: Part I: Review of principles and methods. Clin Nutr.

[16] Janssen I, Heymsfield SB, Baumgartner RN, Ross R (2000). Estimation of skeletal muscle mass by bioelectrical impedance analysis. J Appl Physiol (1985).

[17] Janssen I, Baumgartner RN, Ross R, Rosenberg IH, Roubenoff R (2004). Skeletal muscle cutpoints associated with elevated physical disability risk in older men and women. Am J Epidemiol.

[18] Cruz-Jentoft AJ, Baeyens JP, Bauer JM, Boirie Y, Cederholm T, Landi F (2010). Sarcopenia: European consensus on definition and diagnosis: report of the European Working Group on Sarcopenia in Older People. Age Ageing.

[19] Guralnik JM, Simonsick EM, Ferrucci L, Glynn RJ, Berkman LF, Blazer DG (1994). A short physical performance battery assessing lower extremity function: association with self-reported disability and prediction of mortality and nursing home admission. J Gerontol.

[20] Cesari M, Kritchevsky SB, Newman AB, Simonsick EM, Harris TB, Penninx BW (2009). Added value of physical performance measures in predicting adverse healthrelated events: results from the Health, Aging And Body Composition Study. J Am Geriatr Soc.

[21] Kempen GIJM, Miedema I, Ormel J, Molenaar W (1996). The assessment of disability with the Groningen Activity Restriction Scale: conceptual framework and psychometric properties. Soc Sci Med.

[22] EuroQol Group. EuroQol: a new facility for the measurement of health-related quality of life. Health Policy 1990;16(3):199-208. 10.1016/0168-8510(90)90421-9 10109801

[23] Rabin R, Oemar M, Oppe M, Janssen B, Herdman M (2011). EQ-5D-5L user guide: basic information on how to use the EQ-5D-5L instrument: version 1.0.

[24] Hakkaart-van Roijen L, Tan SS (2011). Bouwmans CAM. Handleiding voor kostenonderzoek: methoden en standaard kostprijzen voor economische evaluaties in de gezondheidszorg: geactualiseerde versie 2010.

[25] De Vries NM, Staal JB, Van der Wees PJ, Adang EM, Akkermans R, Olde Rikkert MG (2016). Patient-centred physical therapy is (cost-) effective in increasing physical activity and reducing frailty in older adults with mobility problems: a randomized controlled trial with 6 months follow-up. J Cachexia Sarcopenia Muscle.

[26] Groessl EJ, Kaplan R C, Sweet CM, Church T, Espeland MA, Gill TM (2016). Cost-effectiveness of the LIFE physical activity intervention for older adults at increased risk for mobility disability. J Gerontol A Biol Sci Med Sci.

[27] Lo YC, Wahlqvist ML, Huang YC (2017). Medical costs of a low skeletal muscle mass are modulated by dietary diversity and physical activity in community-dwelling older Taiwanese: a longitudinal study. Int J Behav Nutr Phys Act.

[28] Bauer JM, Verlaan S, Bautmans I, Brandt K, Donini LM, Maggio M (2015). Effects of a vitamin D and leucine-enriched whey protein nutritional supplement on measures of sarcopenia in older adults, the PROVIDE study: a randomized, double-blind, placebocontrolled trial. J Am Med Dir Assoc.

[29] Yoshimura Y, Uchida K, Jeong S, Yamaga M (2016). Effects of Nutritional Supplements on Muscle Mass and Activities of Daily Living in Elderly Rehabilitation Patients with Decreased Muscle Mass: A Randomized Controlled Trial. J Nutr Health Aging.

[30] Beaudart C, Reginster JY, Petermans J, Gillain S, Quabron A, Locquet M (2015). Quality of life and physical components linked to sarcopenia: the SarcoPhAge study. Exp Gerontol.

[31] Go SW, Cha YH, Lee JA, Park HS (2013). Association between sarcopenia, bone density, and health-related quality of life in Korean men. Korean J Fam Med.

[32] Gobbens R V, Assen MA, Schalk MJ (2014). The prediction of disability by self-reported physical frailty components of the Tilburg Frailty Indicator (TFI). Arch Gerontol Geriatr.

[33] Gani F, Buettner S, Margonis GA, Sasaki K, Wagner D, Kim Y (2016). Sarcopenia predicts costs among patients undergoing major abdominal operations. Surgery.

[34] Kirk PS, Friedman JF, Cron DC, Terjimanian MN, Wang SC, Campbell DA (2015). One-year postoperative resource utilization in sarcopenic patients. J Surg Res.

