# Supplementary material for: Muscle, Health and Costs: A Glance at their Relationship
Source: J Nutr Health Aging. 2018 Jun 13;22(7):766–73. doi: 10.1007/s12603-018-1058-9 (PMC6061527; doi:10.1007/s12603-018-1058-9)
Supplement: Supplementary file 1 — Supplementary Table 1 Spearman correlations (r) between muscle, health and economic outcomes in ‘no care’ group (n = 157) [file 12603_2018_1058_MOESM1_ESM.docx]

**Supplementary Table 1** Spearman correlations (r) between muscle, health and economic outcomes in ‘no care’ group (n = 157)

|  | ADL | |  | Quality of life | |  | Health care costs | |
| --- | --- | --- | --- | --- | --- | --- | --- | --- |
|  | r | P Value |  | r | P Value |  | r | P Value |
| SMI, kg/m^2^ |  |  |  |  |  |  |  |  |
| Men | -0.04 | .71 |  | 0.07 | .52 |  | -0.08 | .45 |
| Women | -0.02 | .89 |  | -0.01 | .91 |  | 0.02 | .86 |
| Grip strength, kg |  |  |  |  |  |  |  |  |
| Men | -0.13 | .23 |  | 0.17 | .12 |  | -0.09 | .42 |
| Women | -0.37 | <.01 |  | 0.34 | <.01 |  | -0.13 | .29 |
| Gait speed, m/s | -0.46 | <.01 |  | 0.34 | <.01 |  | -0.15 | .07 |
| Chair stand, seconds | 0.36 | <.01 |  | -0.23 | <.01 |  | 0.09 | .29 |
| SPPB total, score 0-12 | -0.45 | <.01 |  | 0.26 | <.01 |  | -0.15 | .07 |

ADL, activities of daily living (based on GARS score); SMI, skeletal muscle index; SPPB, short physical performance battery.
